# Surgical pulmonary valve replacement at a tertiary adult congenital heart centre in the current era^☆^

**DOI:** 10.1016/j.ijcchd.2022.100394

**Published:** 2022-05-14

**Authors:** Kana Kubota, Gerhard-Paul Diller, Aleksander Kempny, Andreas Hoschtitzky, Yasushi Imai, Masaaki Kawada, Darryl Shore, Michael A. Gatzoulis

**Affiliations:** a Adult Congenital Heart Centre and National Centre for Pulmonary Hypertension, Royal Brompton Hospital, London, United Kingdom; b Division of Cardiovascular Medicine, Department of Internal Medicine, Jichi Medical University, Tochigi, Japan; c Department of Cardiovascular Medicine, Division of Adult Congenital and Valvular Heart Disease, University Hospital Muenster, Germany; d National Heart and Lung Institute, Imperial College, London, United Kingdom; e Department of Cardiothoracic Surgery and Transplantation, Royal Brompton Hospital, London, United Kingdom; f Division of Clinical Pharmacology, Department of Pharmacology, Jichi Medical University, Tochigi, Japan; g Division of Pediatric and Congenital Cardiovascular Surgery, Department of Surgery, Jichi Medical University, Tochigi, Japan

**Keywords:** Pulmonary valve replacement, Tetralogy of fallot, Adult congenital heart disease

## Abstract

**Background:**

Surgical pulmonary valve replacement (PVR) is the standard cardiac operation in adult patients with congenital heart disease (ACHD). We report recent experience at a large tertiary centre and examine the impact of prosthesis type on outcomes.

**Methods:**

All surgical PVRs performed at our tertiary centre between January 2003 and December 2018 were included.

**Results:**

The study population included 490 patients (197 women; mean age 29.9 ± 13 years). The types of valves used were Homograft 179 (37%), Perimount 152 (31%), Mosaic 120 (24%), Hancock 31 (6%), and others 5 (1%). Seven (1.4%) early deaths occurred within 30 days after surgery. The survival rates at 1, 5, 10, and 15 years of follow-up were 98.3%, 97.7%, 96.8%, and 95.4%, respectively, whereas freedom from re-intervention was 99.8%, 96.6%, 90.2%, and 81.0%, respectively. During a mean follow-up of 6.5 ± 4.3 years, 27 (5.5%) patients required re-intervention. The type of valve used in these 27 patients during index operation showed no discernible difference in the probability of re-intervention; however, Mosaic valves required earlier re-intervention compared to Perimount valves.

**Conclusion:**

Our data show no discernible difference in reoperation rates between the different types of prostheses used. On-going surveillance of patients after PVR and further research in developing a life-long prosthesis are clearly warranted.

## 1 Introduction

Surgical pulmonary valve replacement (PVR) is the most common cardiac operation in adult patients with congenital heart disease (ACHD) [1]. PVR has been employed as part of repair of tetralogy of Fallot (TOF) with pulmonary stenosis or atresia for more than 50 years [2,3]. Since the beginning of this era, when human homografts were used, a number of bioprosthetic valves have been developed and implanted in the pulmonary position. Furthermore, percutaneous PVR is now established as a surgical alternative, particularly for patients with previous homograft conduit repair. Several reports have demonstrated the positive impact of PVR, including marked reduction of pulmonary regurgitation (PR), reduction in right ventricle (RV) volumes, improved LV filling, decrease in RV systolic pressure, and improved functional class; thus, PVR is here to stay [[4], [5], [6], [7]]. However, the functional integrity of all bioprosthetic valves in the pulmonary position deteriorates over time in patients requiring repeated PVR within a decade or two. Strategies and techniques of PVR have steadily evolved with the aim of improving long-term results [[8], [9], [10], [11]]. However, despite numerous reports on timing, technique, and results of PVR, limited information is available on the varying durability of bioprosthetic valves in the pulmonary position in the current era. The aim of our study was to examine the outcome of PVR and the impact of the type of valve prosthesis on the need for reoperation at a high-volume tertiary ACHD centre.

## 2 Methods

### 2.1 Patients

All patients (over 12 years old) who underwent surgical PVR between January 2003 and December 2018 at the Royal Brompton Hospital (RBH), London, UK, were included. Patients were identified from our surgical database, and their hospital records were examined. As this was a retrospective analysis of data collected for routine clinical care, individual informed consent was not required (UK National Research Ethics Service guidance). This study was registered and approved locally.

From a total of 647 PVRs performed during the study period, we excluded patients undergoing non-isolated PVR (Ross procedure [115], complete transposition of the great arteries [29], congenitally corrected transposition of the great arteries [truncus arteriosus {4}, ventricular septal defect {3}, double inlet right ventricle {1}, and anomalous left coronary artery from the pulmonary artery {1}]).

Data recorded included demographics, information on previous surgical palliation and repair, preoperative New York Heart Association (NYHA) class, indication for PVR, details of the surgical procedure including the type and size of valve prosthesis used. Regarding right ventricular function, we analysed data in all cases where transthoracic echocardiography and cardiac magnetic resonance imaging (CMR) were available.

At the Royal Brompton Hospital, we investigated the trends of PVR, and further mortality and all-cause re-intervention, whether surgical or percutaneous.

### 2.2 Statistical analysis

Quantitative data are shown as the mean ± SD or percentage. Student's t-test and categorical variables were compared using the chi-squared test between the groups. Kaplan-Meier curves were constructed to illustrate survival and freedom from events, and log-rank P-values were provided. All statistical analyses were performed using SPSS Statistics (version 25.0; IBM Corp., Armonk, NY, USA), while Kaplan-Meier curves were plotted using EZR version 1.40 (Saitama Medical Center, Jichi Medical University, Saitama, Japan) [12]. Statistical significance was set at P < 0.05.

## 3 Results

The patient characteristics are summarised in Table 1. Between January 2003 and December 2018, 490 patients including 197 (40%) women who underwent PVR, constituted our study population. The mean age at index PVR was 29.9 ± 12.8 (range, 12–71) years. The primary diagnoses were TOF (358, 74%), pulmonary stenosis (PS) (96, 20%), pulmonary atresia (28, 6%), right ventricular outflow tract (RVOT) obstruction (3, 0.6%), PR (3, 0.6%), absent pulmonary valve (1, 0.2%), and other pulmonary valve abnormalities (1, 0.2%). A total of 478 patients (98%) had a previous intervention, most commonly TOF repair (345, 70%). 77 patients underwent initial open pulmonary valvotomy, 21 had balloon pulmonary valvotomy, 15 had RV- pulmonary artery (PA) conduit without TOF, nine had surgical PVR, five had percutaneous PVR, three had RVOT relief procedure, two had radiofrequency pulmonary valvotomy and one had supravalvular PS. The majority of patients (76%) were NYHA class I. 452 (93%) patients underwent surgery with PR as an indication, 28 patients had PS, and 10 patients had infective endocarditis (IE) as an indication for PVR.

**Table 1:** Characteristics of PVR patients.

Characteristics	n = 490
Age, y	30.0 ± 12.8
Female, %	40.2
**Diagnosis**	
TOF	358
PS	96
Pulmonary atresia	28
RVOT obstruction	3
PR	3
Absent pulmonary artery	1
Pulmonary valve abnormality	1
**Previous Intervention**	
Number of previous intervention	1.06 ± 0.36
TOF repair	345
Open pulmonary valvotomy	77
Balloon pulmonary valvotomy	21
RV-PA conduit	15
Surgical PVR	9
Percutaneous PVR	5
RVOT relief	3
Radiofrequency pulmonary valvotomy	2
Supravalvar PS relief	1
None	12
**NYHA Classification**	
1	369
2	85
3	0
4	0
No data	36
**Indication for Surgery**	
PR	452
PS	28
IE	10
**Surgical data**	
Total bypass time, mins	105.9 ± 64.1
Total cross clamp time, mins	40.5 ± 40.0

Data are mean ± SD or percentage. TOF indicates tetralogy of Fallot; PS, pulmonary stenosis; RVOT, right ventricular outflow tract; PR, pulmonary regurgitation; RV-PA, right ventricle -pulmonary artery; PVR, pulmonary valve replacement; NYHA, New York Heart Association; IE, infective endocarditis.

The total bypass time for the index PVR was 105.9 ± 64.1 min, whereas the total cross-clamp time was 40.5 ± 40.0 min. The parameters for right heart assessment are shown in Table 2. Echocardiography and CMR were performed preoperatively and postoperatively in 176 and 150 patients, respectively. Postoperative echocardiography and CMR were performed within two years after surgery. Pre-operative pulmonary valve velocity was 237.7 ± 99.6 cm/s and it declined to 214.3 ± 53.3 cm/s after PVR. There were 161 patients who had at least moderate PR before PVR and only five patients had persistent moderate or more PR postoperatively. One of the five cases underwent re-intervention due to IE, but no obvious reasons why they developed further PR in the remaining four cases. Pre-operative RV end-diastolic volume (RVEDV) index and RV end-systolic volume (RVESV) index were 159.2 ± 38.7 mL and 85.2 ± 30.6 mL/m^2^, and postoperatively, they improved to 106.9 ± 23.1 mL and 55.2 ± 19.0 mL/m^2^, respectively.

**Table 2:** Parameters for right heart assessment.

Echocardiography	All patients n = 176	TOF n = 121	PS n = 40	P value
Pre-operative pulmonary valve velocity, m/s	2.4 ± 0.9	2.4 ± 0.9	2.3 ± 1.1	0.526
Pre-operative pulmonary regurgitation				
No	7	2	3	
Trivial	0	0	0	
Mild	6	3	3	
Moderate	18	13	4	
Severe or free	143	101	30	
No data	3	2	0	
Post-operative pulmonary valve velocity, m/s	2.1 ± 0.5	2.2 ± 0.5	2.0 ± 0.5	0.185
Post-operative pulmonary regurgitation				
No	71	44	19	
Trivial	57	46	9	
Mild	42	27	10	
Moderate	4	2	2	
Severe or free	1	1	0	
No data	2	1	0	
**CMR**	**All patients n** = **150**	**TOF n** = **109**	**PS n** = **27**	
Pre-operative RVEDV index, ml/m^2^	159.2 ± 38.7	159.7 ± 34.6	155.8 ± 40.9	0.148
Pre-operative RVESV index, ml/m^2^	85.2 ± 30.6	86.0 ± 29.4	79.4 ± 31.1	0.061
Post-operative RVEDV index, ml/m^2^	106.9 ± 23.1	106.2 ± 23.0	109.1 ± 22.3	0.136
Post-operative RVESV index, ml/m^2^	55.2 ± 19.0	56.7 ± 20.0	52.4 ± 13.3	0.006

Data are mean ± SD or number. TOF indicates tetralogy of Fallot; PS, pulmonary stenosis; CMR, cardiac magnetic resonance imaging; RVEDV, right ventricle end-diastolic volume; RVESV, right ventricle end-systolic volume.

### 3.1 Type of valve prosthesis

**Fig. 1:**
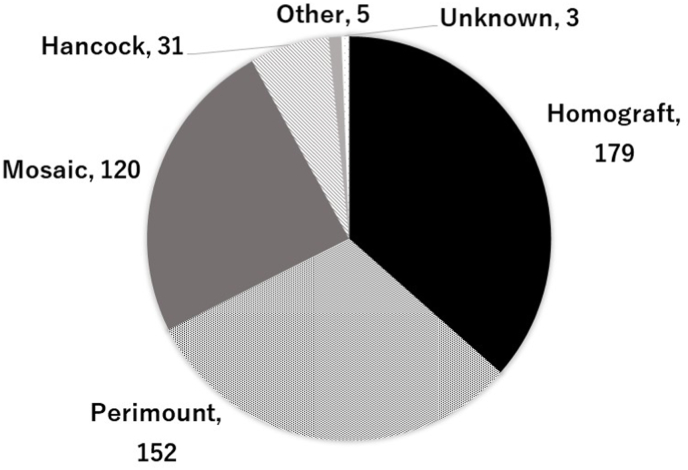
The types of valve prosthesis.

**Fig. 2:**
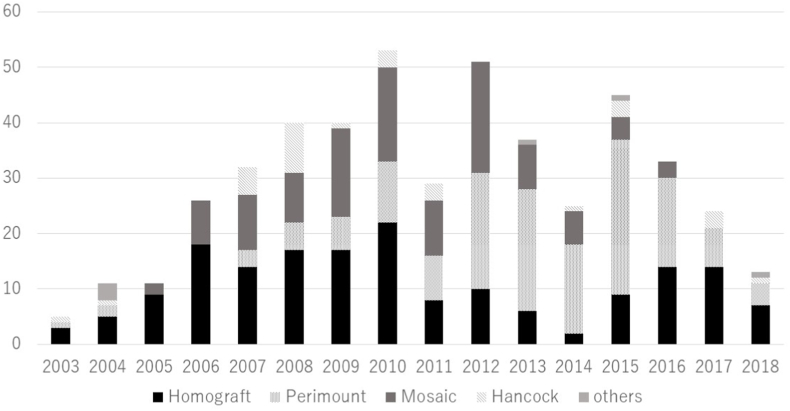
Choice of valve type over time.

The types of valve prostheses employed are shown in Fig. 1. The prostheses used were Homograft in 179 patients (37%), Perimount (Edwards Lifesciences, Irvine, CA) in 152 (31%), Mosaic (Medtronic Inc.; Minneapolis, MN) in 120 (24%), Hancock (Medtronic Inc.; Minneapolis, MN) in 31 (6%), and others in five patients (1%). Of the others, three patients received a Mitraflow (Sorin Group Inc.; Vancouver, Canada), one received a Magna (Edwards Lifesciences, Irvine, CA), and one St. Jude Medical (SJM) valve (St. Jude Medical Inc.; Minnesota, MN). Three patients did not have any information regarding the type of prosthesis. The choice of valve type over time revealed a trend towards an increased use of stented bioprostheses from 2003 to 2015, with a more recent decline in the utilisation of Mosaic valves at our centre (Fig. 2). Furthermore, the number of surgical PVRs has been declining due to the increasing use of percutaneous PVR.

### 3.2 Mortality

**Fig. 3:**
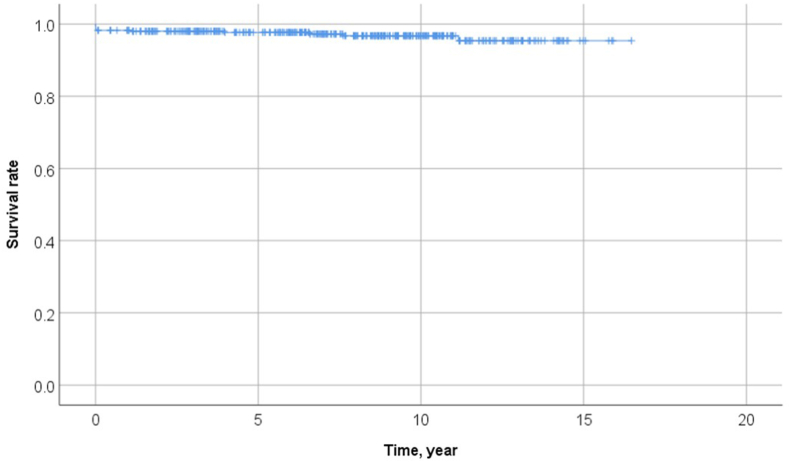
Kaplan-Meier curve of mortality.

The primary outcomes are shown in Table 3. There were seven (1.4%) early deaths within 30 days of surgery; patients had a homograft (4), Mosaic (1), Perimount (1), and unknown in one. There were five late deaths, two of which were cardiac-related. Fig. 3 shows the Kaplan-Meier survival curves after PVR. The survival rates at 1, 5, 10, and 15 years were 98.3%, 97.7%, 96.8%, and 95.4%, respectively.

**Table 3:** Mortality after PVR.

Early mortality, n = 7 (1.4%)						
Age/Sex		Diagnosis	Previous procedure	Valve	Cause of death	
42 F		TOF	TOF repair	No data	No data	
36 F		TOF	TOF repair	Aortic homograft	Bleeding	
34 M		TOF	TOF repair	Pulmonary homograft	No data	
30 M		TOF	TOF repair	Aortic homograft	IE	
29 M		TOF	Percutaneous PVR	Aortic homograft	Injured LCA during procedure	
50 M		TOF	TOF repair	Mosaic	VT/VF	
47 M		PS	Pulmonary valvotomy	Perimount	Injured RCA during procedure	

**Late mortality, n** = **5**						
**Age/Sex**	**Diagnosis**		**Previous procedure**	**Valve**	**Cause of death**	**Time from index PVR (year)**
18 M	TOF		TOF repair	Mosaic	Pneumonia	6.6
51 M	TOF		TOF repair	Aortic homograft	Breast cancer	7.6
17 M	TOF		TOF repair	Perimount	No data	4.0
57 F	TOF		TOF repair	Hancock	Heart failure	11.2
56 M	RVOTO		RVOT procedure	Perimount	VT	1.1
PVR indicates pulmonary valve replacement; TOF, tetralogy of Fallot; PS, pulmonary stenosis; RVOT, right ventricular outflow tract; RVOTO, right ventricular outflow tract obstruction; IE, infective endocarditis, LCA, left coronary artery; RCA, right coronary artery; VT, ventricular tachycardia; VF, ventricular fibrillation.

### 3.3 Re-intervention

The secondary outcome of the re-intervention is shown in Table 4. During a mean follow-up of 6.5 ± 4.3 years, 27 (5.5%) patients required re-intervention after index PVR. Table 4 also shows the characteristics of patients requiring valvular re-intervention according to the type of valve used (12 patients initially had a homograft [44%], 11 a Mosaic valve [41%], two a Perimount valve [7%], and two a Hancock valve [7%]). Indications for re-intervention were IE in 10 patients (the details are shown in supplemental table 1), PR in eight patients, PS in eight cases, and other in one. Distal obstruction of the pulmonary arteries is considered to be one of the factors which may lead to early valve failure. We analysed the cases with a history of previous PA stenting or ballooning or the need for concomitant PA angioplasty at the re-intervention. Distal obstruction of PA was suspected in 3 of the 27 patients who required re-intervention after PVR but there was no significant difference in the no with distal obstruction compared to the group that did not require re-intervention. The time from index surgery to re-operation was 6.1 ± 3.4 years. The types of valves employed for re-intervention were 16 surgical, 9 percutaneous, 1 balloon valvuloplasty, and 1 heart transplantation (patients with TOF developing “cardiomyopathy” and severe biventricular failure after index PVR).

**Table 4:** Comparison between patients regarding re-intervention and the remainder.

Characteristics	n = 27	n = 463	P value
Age at index PVR, y	24.6 ± 10.7	30.2 ± 12.8	0.026
Female, %	33.3	40.6	0.454
**Follow up**			
Time, y	9.6 ± 3.9	6.2 ± 4.2	<0.001
**Diagnosis**			
TOF	18	340	0.298
PS	6	90	0.485
Pulmonary atresia	3	25	0.202
RVOT obstruction	0	3	0.733
PR	0	3	0.619
Absent pulmonary valve	0	1	0.656
Pulmonary valve abnormality	0	1	0.962
**Previous Intervention**			
Number of previous interventions	0.9 ± 0.4	1.1 ± 0.4	0.012
TOF repair	17	328	0.185
Open pulmonary valvotomy	4	73	0.981
Balloon pulmonary valvotomy	0	21	0.550
RV-PA conduit	1	14	0.982
PVR	1	8	0.182
Percutaneous PVR	0	5	0.719
RVOT relief	0	3	0.681
Radiofrequency pulmonary valvotomy	0	2	0.802
supravalvar PS relief	0	1	0.962
None	4	8	0.001
**NYHA Classification**			
1	21	348	0.616
2	3	82	0.748
3	0	0	–
4	0	0	–
No data	3	33	0.735
**Indication for index PVR**			
PR	15	437	–
PS	9	19	–
IE	3	7	–
**Surgical information**			
Total bypass time, mins	146.4 ± 120.9	103.5 ± 58.3	0.084
Total cross clamp time, mins	31.7 ± 41.0	41.0 ± 40.0	0.245
Valve size	24.3 ± 2.0	24.9 ± 1.9	0.123
**Type of valve at the index operation**			
Homograft, n (%)	12 (44.4)	167 (36.1)	0.625
Mosaic, n (%)	11 (40.7)	109 (23.5)	0.203
Perimount, n (%)	2 (7.4)	150 (32.4)	0.067
Hancock, n (%)	2 (7.4)	29 (6.3)	0.832
Others, n (%)	0	8 (1.7)	0.416
**Concomitant PA surgery or pre PA stenting**			
Yes	3 (11.1)	74 (16.0)	–
No	24 (88.8)	389 (84.0)	–
**Surgeon at the index operation**			
Surgeon A, n (%)	6 (22.2)	196 (42.3)	–
Surgeon B, n (%)	9 (33.3)	103 (22.2)	–
Surgeon C, n (%)	6 (22.2)	63 (13.6)	–
Others, n (%)	6 (22.2)	101 (21.8)	–
**Indication for re-intervention**			
IE	10	NA	–
Homograft	4		
Mosaic	3		
Perimount	2		
Hancock	1		
PR	8	NA	–
Homograft	4		
Mosaic	4		
Perimount	0		
Hancock	0		
PS	8	NA	–
Homograft	4		
Mosaic	3		
Perimount	0		
Hancock	1		
Other	1 (Mosaic)	NA	–
**Time until re-intervention**			
Time, y	6.1 ± 3.4	NA	–
**Type of re-intervention**			
PVR	16	NA	–
Percutaneous PVR	9	NA	–
Balloon valvuloplasty	1	NA	–
Other	1 (Heart transplantation)	NA	–

Data are mean ± SD or number. TOF indicates tetralogy of Fallot; PS, pulmonary stenosis; RVOT, right ventricular outflow tract; PR, pulmonary regurgitation; RV-PA, right ventricle -pulmonary artery; PVR, pulmonary valve replacement; NYHA, New York Heart Association; PA, pulmonary artery; IE, infective endocarditis.

**Fig. 4:**
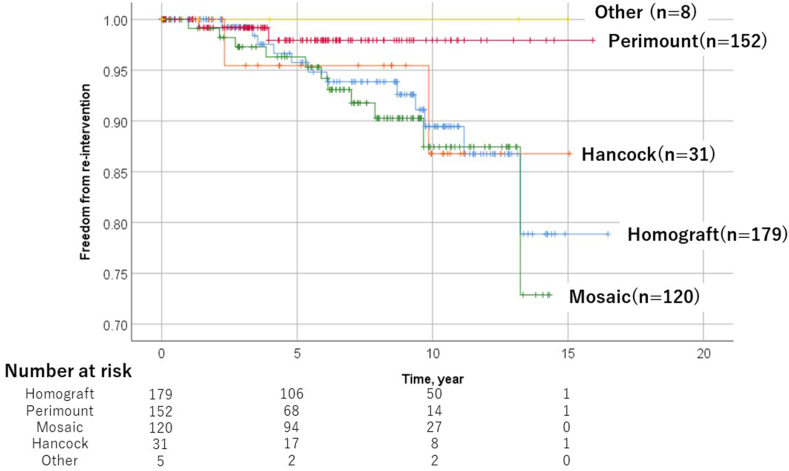
Kaplan-Meier curve of freedom from re-intervention divided by type of prosthesis.

Fig. 4 illustrates the Kaplan-Meier re-interventional curves after PVR, according to the type of valve prosthesis employed. The freedom rates from re-intervention at 1, 5, 10, and 15 years were 100%, 95.8%, 89.5%, and 78.9%, respectively. Among the patients with Perimount prosthesis, the rates of reintervention at 1, 5, 10, and 15 years were 100%, 97.9%, 97.9%, and 97.9%, respectively. The freedom rates from re-intervention for 1, 5, 10, and 15 years were 99.1%, 96.3%, 87.4%, and 72.9%, respectively, in the Mosaic group. Patients receiving Hancock prosthesis showed a freedom rate from re-intervention for 1, 5, 10, and 15 years of 100%, 95.5%, 86.8%, and 86.8%, respectively, with no difference between them. Mosaic valves tended to require earlier re-intervention compared to the Perimount valves (*p* = 0.047, log-rank test).

Implantation of a homograft for PVR can be associated with early pulmonary regurgitation due to technical error. It important to correct the length of the homograft to avoid kinking and remodel the RVOTO in order to match the size to the homograft at the proximal anastomosis at the proximal anastomosis. We analysed the outcomes of three main surgeons who used a homograft in 10 or more cases, but no significant difference in outcomes was observed.

## 4 Discussion

In our single-centre tertiary cohort reflecting a 15-year experience of surgical PVR in 490 patients, the main findings can be summarised as follows: (1) Despite the heterogeneous and increasingly challenging patient population, isolated pulmonary valve replacement is a low-risk procedure with early mortality rates of no more than 1.4%; (2) the probability of re-intervention at 5, 10, and 15 years of follow-up was low at 3.4%, 9.8%, and 19.0%, overall; (3) there were no clear, discernible differences regarding the need for re-intervention based on the type of PVR prostheses employed, albeit there was a trend regarding Mosaic prostheses requiring earlier re-intervention compared to, for example, Perimount valves.

### 4.1 Timing of PVR in ACHD patients

Since bioprosthetic valves have a limited life span of approximately 10–15 years, PVR in ACHD warrants careful consideration regarding patient selection, timing, and valve selection. Regarding TOF, in particular, there are numerous reports on the timing of the intervention. The European Society of Cardiology (ESC) and the American College of Cardiology/American Heart Association have published independent recommendations for PVR in adults with repaired TOF [13,14]. Although there are some differences between the two guidelines, there is consensus that the combination of severe PR and/or symptoms (diminished exercise capacity, dyspnoea, new-onset of clinical arrhythmia), and/or early heart failure constitutes indications for PVR. Guidelines also suggest that adults with severe PR and moderate-to-severe RV enlargement may be considered for PVR. ESC guidelines suggests that PVR should be considered in asymptomatic patients with severe PR and/or RVOT) obstruction, in the presence of progressive RV dilation (RVESV index ≥80 mL/m^2^, and/or RVEDV index ≥160 mL/m^2^) and/or progression to at least moderate TR [13]. The Canadian guidelines also suggests an RVEDV index >170 mL/m^2^ is the threshold to consider PVR [15]. In our study 150 patients underwent preoperative and postoperative CMR, of which 109 had a preoperative RVESV index of 85.2 ± 30.6 mL/m^2^ and RVEDV index of 159.7 ± 34.6 mL/m^2^. RVEDV value was lower than the threshold from the guideline. However, from the perspective of RVESV, it was a surgical indication. This RVESV standard suggests a more proactive approach for PVR before right ventricular dilatation and associated dysfunction or clinical decomposition ensue, based on our previous report [16]. In the beginning of our experience, we saw more symptomatic patients. Recently, we are largely guided by non-invasive (CMR and echo) assessment of RV function and volumes, using these indications for PVR. Residual ventricular septal defects and/or aortic root dilation, aortic regurgitation should also be addressed at the time of surgery [13]. Conversely, there is limited information about the residual PR after repair of congenital PS. There is data that postponing PVR until symptoms or progressive RV dilation may be considered in residual PR of PS patients, because a more robust improvement of RV hemodynamic parameters can be expected when compared with matched TOF patients [17]. We evaluated preoperative and postoperative CMR in 27 PS patients, and the preoperative RVEDV was 155.8 ± 40.9 mL/m^2^, indicating that surgery is likely to be performed according to the surgical indication criteria for TOF patients with PR.

### 4.2 Mortality related to PVR

PVR in adults with repaired TOF has a low early mortality risk (0–4.6%), with the majority of patients being free of major postoperative complications [[18], [19], [20], [21]]. Similar to these reports, the early mortality in our study was low at 1.4%. Two patients died of intraoperative coronary artery kinking. Dos et al. pointed out that patients with TOF have a high incidence of myocardial infarction during the perioperative period of PVR [20]. Because between 2% and 10% of patients with TOF have anomalous coronary arteries crossing the RVOT, the anomalous arteries might pose problems at the time of intracardiac repair, even when identified preoperatively. These abnormal coronary arteries are more likely to be damaged by repeat operations and adhesions, which create additional difficulties in the surgical field. More detailed screening of coronary artery anatomy with preoperative imaging may prevent intraoperative coronary artery injury.

Similar to our results, previous reports have reported a 10-year survival rate of 92%–95% for ACHD patients undergoing PVR after previously corrected TOF [20,22,23]. In our current study, one in each of the early and late deaths was due to VT. Ventricular arrhythmias are known to be an important factor in long-term morbidity and mortality in patients with TOF, whereas sudden cardiac death (SCD) with an incidence of up to 6% has been reported in some long-term follow-up studies [24]. It is hoped that earlier PVR and improved haemodynamics may reduce the incidence of SCD, although this is speculative at present. Furthermore, improved PVR strategies for SCD in corrected TOF and catheter ablation and/or ICD implantation are likely to reduce SCD in these patients.

### 4.3 Types of prosthesis for PVR

The vast majority of prostheses used for PVR are homografts or valved xenografts because insufficient data are available to date on right-sided mechanical valves. The risk of thrombosis is considered to be high [[25], [26], [27]], necessitating life-long anticoagulation for metallic valves in this setting with difficulty in managing low-pressure circulation [28]. In our study, a metallic prosthesis was used only in one patient (SJM valve). In recent years, however, with the evolution of mechanical valve design, there have been a series of reports where valvular insufficiency due to thrombus formation and/or reoperations can be avoided with strictest anticoagulant regimes [[29], [30], [31]].

The most frequently used prosthesis in our study was a homograft. Aortic and pulmonary cryopreserved homografts have been in use. A 5-year freedom from homograft failure was better for pulmonary homografts compared to aortic homografts according to the literature [32]. Various factors, such as size of valve prostheses, recipient age, and extra-anatomical use influence homograft durability; while, younger age of homograft donors, later year of operation, ABO blood group incompatibility, stenosis of the pulmonary arteries, and associated absent pulmonary valve syndrome may be associated with earlier homograft dysfunction [33,34].

All currently available bioprostheses have relatively favourable haemodynamic properties and do not require anticoagulation therapy. However, in the medium-to-long term, calcification occurs at a high rate, and 10-year freedom from re-intervention remains at approximately 50%–70% [35,36]. Perimount is a stented valve made from bovine pericardium that performs well on freedom from re-intervention [37,38]. The Hancock prosthesis, also a stent valve, made from a porcine aortic valve has a similar good record and has been widely used for PVR [39]. Mosaic is a third-generation stented valve made from porcine aorta with a flexible stent similar to Hancock and anti-calcification tissue treatment similar to Freestyle. It has been reported that Mosaic valves have the same 3-year freedom from re-intervention as the Perimount and homograft valves in the pulmonary position [40].

There were some changes in the choice of prosthesis used in the course of our study. This may reflect the limited availability of suitably sized valves (most homografts) and/or a change in surgical preference. The vast majority of PVRs at the RBH during the study period were performed by three surgeons. Choice of prostheses varied between the three and was influenced by personal and previous institutional experience, the perceived difficulty of surgery, the no of previous operations and the anatomy of the pulmonary arteries and RVOTO. The ease of percutaneous PVR at subsequent re-intervention the religion and preference of the patients might also be factored in. However, all the surgeons were experienced and we have no evidence to suggest that differences in technique affected outcome. Additionally, the reduced use of porcine valves may be partly due to some data suggesting that Mosaic valves were more prone to deterioration than bovine valves and had higher redo ratios or require earlier redo PVRs [[41], [42], [43]].

### 4.4 Surgical approach in PVR

Unfortunately, there was no protocol shared between surgeons in RBH in terms of the surgical approach in PVR, so the surgeons each had their individual approach. At the beginning of the study period, surgeon no1 (operated on 202 of the 490 cases in our study) preferred to perform the PVR with the heart beating. However, he would currently reserve this technique for patients with poor ventricular function. We see fewer of these patients now than at the beginning of our experience. When performing PVR with a beating heat, it is essential to exclude an intra cardiac shunt. The vast majority of cases he now performs using cardioplegic arrest with body temperature at 32 °C. Institution of Femoral -femoral bypass pre sternotomy is reserved for those patients in which the heart or great vessels are closely applied to the inner table of the sternum on MRI and CT.

### 4.5 Re-intervention after PVR

We previously reported freedom from redo PVR in our adult series of 100% at 1 year, 98% at 5 years, and 96% at 10 years [44]. The 10-year freedom from re-intervention in other reports ranged from 69% to 85% [21,[45], [46], [47]]. In previous studies, numbers seem to have varied in the mixed population of children and adults; clearly, children are susceptible to earlier reoperation due to somatic growth and associated valve degeneration [48]. In our current study, the freedom rate from re-intervention at 1, 5, 10, and 15 years were 99%, 96%, 90%, and 81%, respectively, which is in agreement with previous reports. Regarding the types of prosthesis used, our data showed no discernible differences in the probability of re-intervention for PVR, although Mosaic valves tended to require earlier re-intervention compared to Perimount valves. Fiore and colleagues compared outcomes of three types of prostheses: porcine valve (Mosaic), bovine pericardial valve (Perimount), and pulmonary homografts [40]. They reported that the 5-year freedom from re-intervention was 92% for the Perimount group, compared to 78% for the Mosaic and pulmonary homograft groups. Additionally, there are data showing better midterm durability of pericardial versus porcine valves [49]. Structural valve deterioration seems to begin at 7–8 years with porcine valves and 11–12 years with pericardial valves [50]. Our results are consistent with these reports and were attributed to the rapid deterioration of porcine valves.

The most frequent indication for redo PVR in our series was IE, admittedly follow up of 6.5 ± 4.3 years being not long enough to evaluate complete valve deterioration with time.

### 4.6 New prosthetic valves for PVR

New prosthetic valves are being developed to overcome xenograft or homograft degeneration, in which is the major cause of patient re-operation after PVR. One of them is the tissue-engineered heart valve (TEHV). TEHV represents an upcoming alternative source to create viable, nonimmunogenic, and biologically active grafts. The concept of TEHV is based on the decellularisation of biological scaffolds, such as xenogeneic and allogeneic heart valves, whether with pre-seeding stem cells or not [51]. Many different protocols for decellularisation of biological sources are being developed, as well as various concepts for preservation after processing [[52], [53], [54]]. TEHV is hoped to allow various valve sizes, accommodating growth, thus potentially applicable to children and young adults. A characteristic handmade valve called expanded polytetrafluoroethylene valved conduit has also been reported in Japan [55]. The characteristic combination of a bulging sinus and a fan-shaped valve realised superior valve function to prevent pulmonary regurgitation (and stenosis), with the potential for better long-term outcomes even among infants and young children [56].

### 4.7 Limitations

There are some limitations to our study. First, it had a retrospective design. Second, considering the lifespan of the bioprosthesis in the pulmonary position, the follow-up period was relatively short. However, we were keen to report our experience as there have been concerns regarding PVR and early valve failures. Furthermore, there is scope to revisit our cohort every five years, and thus we can report future outcomes. Nevertheless, we report the largest (n = 490), single-centre, unselected clinical series with a complete data set on early mortality and the need for re-intervention. Outcomes from our tertiary centre with high congenital heart disease volumes cannot necessarily be extrapolated to other settings. However, we submit that our group of patients and reported approach to be representative of contemporary tertiary ACHD practice.

## 5 Conclusion

Surgical PVR is associated with lower early mortality (1.4%) and a probability of re-intervention of 3.4%, 9.8%, and 19.0% at 5, 10, and 15 years, respectively. Our data show no discernible difference in reoperation rates between the different types of prostheses used. Ongoing surveillance of patients after PVR and further research in developing a life-long prosthesis are clearly warranted.

## Any potential conflicts of interest, including related consultancies, shareholdings and funding grants

None.
