# Supplementary material for: Surgical pulmonary valve replacement at a tertiary adult congenital heart centre in the current era
Source: Int J Cardiol Congenit Heart Dis. 2022 May 14;9:100394. doi: 10.1016/j.ijcchd.2022.100394 (PMC11657547; doi:10.1016/j.ijcchd.2022.100394)
Supplement: Multimedia component 1 [file mmc1.docx]

Table 1. Characteristics of PVR patients

| **Characteristics** | **n=490** |
| --- | --- |
| Age, y | 30.0 ± 12.8 |
| Female, % | 40.2 |
| **Diagnosis** |  |
| TOF | 358 |
| PS | 96 |
| Pulmonary atresia | 28 |
| RVOT obstruction | 3 |
| PR | 3 |
| Absent pulmonary artery | 1 |
| Pulmonary valve abnormality | 1 |
| **Previous Intervention** |  |
| Number of previous intervention | 1.06 ± 0.36 |
| TOF repair | 345 |
| Open pulmonary valvotomy | 77 |
| Balloon pulmonary valvotomy | 21 |
| RV-PA conduit | 15 |
| Surgical PVR | 9 |
| Percutaneous PVR | 5 |
| RVOT relief | 3 |
| Radiofrequency pulmonary valvotomy | 2 |
| Supravalvar PS relief | 1 |
| None | 12 |
| **NYHA Classification** |  |
| 1 | 369 |
| 2 | 85 |
| 3 | 0 |
| 4 | 0 |
| No data | 36 |
| **Indication for Surgery** |  |
| PR | 452 |
| PS | 28 |
| IE | 10 |
| **Surgical data** |  |
| Total bypass time, mins | 105.9 ± 64.1 |
| Total cross clamp time, mins | 40.5 ± 40.0 |
| Data are mean±SD or percentage. TOF indicates tetralogy of Fallot; PS, pulmonary stenosis; RVOT, right ventricular outflow tract; PR, pulmonary regurgitation; RV-PA, right ventricle -pulmonary artery; PVR, pulmonary valve replacement; NYHA, New York Heart Association; IE, infective endocarditis. | |

Table 2. Parameters for right heart assessment

| **Echocardiography** | **All patients**  **n=176** |  | **TOF**  **n=121** | **PS**  **n=40** | **P value** |
| --- | --- | --- | --- | --- | --- |
| Pre-operative pulmonary valve velocity, m/s | 2.4 ± 0.9 |  | 2.4 ± 0.9 | 2.3 ± 1.1 | 0.526 |
| Pre-operative pulmonary regurgitation |  |  |  |  |  |
| No | 7 |  | 2 | 3 |  |
| Trivial | 0 |  | 0 | 0 |  |
| Mild | 6 |  | 3 | 3 |  |
| Moderate | 18 |  | 13 | 4 |  |
| Severe or free | 143 |  | 101 | 30 |  |
| No data | 3 |  | 2 | 0 |  |
| Post-operative pulmonary valve velocity, m/s | 2.1 ± 0.5 |  | 2.2 ± 0.5 | 2.0 ± 0.5 | 0.185 |
| Post-operative pulmonary regurgitation |  |  |  |  |  |
| No | 71 |  | 44 | 19 |  |
| Trivial | 57 |  | 46 | 9 |  |
| Mild | 42 |  | 27 | 10 |  |
| Moderate | 4 |  | 2 | 2 |  |
| Severe or free | 1 |  | 1 | 0 |  |
| No data | 2 |  | 1 | 0 |  |
| **CMR** | **All patients**  **n=150** |  | **TOF**  **n=109** | **PS**  **n=27** |  |
| Pre-operative RVEDV index, ml/m^2^ | 159.2 ± 38.7 |  | 159.7 ± 34.6 | 155.8 ± 40.9 | 0.148 |
| Pre-operative RVESV index, ml/m^2^ | 85.2 ± 30.6 |  | 86.0 ± 29.4 | 79.4 ± 31.1 | 0.061 |
| Post-operative RVEDV index, ml/m^2^ | 106.9 ± 23.1 |  | 106.2 ± 23.0 | 109.1 ± 22.3 | 0.136 |
| Post-operative RVESV index, ml/m^2^ | 55.2 ± 19.0 |  | 56.7 ± 20.0 | 52.4 ± 13.3 | 0.006 |
| Data are mean±SD or number. TOF indicates tetralogy of Fallot; PS, pulmonary stenosis; CMR, cardiac magnetic resonance imaging; RVEDV, right ventricle end-diastolic volume; RVESV, right ventricle end-systolic volume. | | | | | |

Table 3. Mortality after PVR

| **Early mortality, n=7 (1.4 %)** | | | | | | |
| --- | --- | --- | --- | --- | --- | --- |
| **Age/Sex** | | **Diagnosis** | **Previous procedure** | **Valve** | **Cause of death** | |
| 42 F | | TOF | TOF repair | No data | No data | |
| 36 F | | TOF | TOF repair | Aortic homograft | Bleeding | |
| 34 M | | TOF | TOF repair | Pulmonary homograft | No data | |
| 30 M | | TOF | TOF repair | Aortic homograft | IE | |
| 29 M | | TOF | Percutaneous PVR | Aortic homograft | Injured LCA during procedure | |
| 50 M | | TOF | TOF repair | Mosaic | VT/VF | |
| 47 M | | PS | Pulmonary valvotomy | Perimount | Injured RCA during procedure | |
|  | | | | | | |
| **Late mortality, n=5** | | | | | | |
| **Age/Sex** | **Diagnosis** | | **Previous procedure** | **Valve** | **Cause of death** | **Time from index PVR (year)** |
| 18 M | TOF | | TOF repair | Mosaic | Pneumonia | 6.6 |
| 51 M | TOF | | TOF repair | Aortic homograft | Breast cancer | 7.6 |
| 17 M | TOF | | TOF repair | Perimount | No data | 4.0 |
| 57 F | TOF | | TOF repair | Hancock | Heart failure | 11.2 |
| 56 M | RVOTO | | RVOT procedure | Perimount | VT | 1.1 |
| PVR indicates pulmonary valve replacement; TOF, tetralogy of Fallot; PS, pulmonary stenosis; RVOT, right ventricular outflow tract; RVOTO, right ventricular outflow tract obstruction; IE, infective endocarditis, LCA, left coronary artery; RCA, right coronary artery; VT, ventricular tachycardia; VF, ventricular fibrillation. | | | | | | |

Table 4. Comparison between patients regarding re-intervention and the remainder

| **Characteristics** | **n=27** | **n=463** | **P value** |
| --- | --- | --- | --- |
| Age at index PVR, y | 24.6 ± 10.7 | 30.2 ± 12.8 | 0.026 |
| Female, % | 33.3 | 40.6 | 0.454 |
| **Follow up** | | | |
| Time, y | 9.6 ± 3.9 | 6.2 ± 4.2 | < 0.001 |
| **Diagnosis** | | | |
| TOF | 18 | 340 | 0.298 |
| PS | 6 | 90 | 0.485 |
| Pulmonary atresia | 3 | 25 | 0.202 |
| RVOT obstruction | 0 | 3 | 0.733 |
| PR | 0 | 3 | 0.619 |
| Absent pulmonary valve | 0 | 1 | 0.656 |
| Pulmonary valve abnormality | 0 | 1 | 0.962 |
| **Previous Intervention** | | | |
| Number of previous interventions | 0.9 ± 0.4 | 1.1 ± 0.4 | 0.012 |
| TOF repair | 17 | 328 | 0.185 |
| Open pulmonary valvotomy | 4 | 73 | 0.981 |
| Balloon pulmonary valvotomy | 0 | 21 | 0.550 |
| RV-PA conduit | 1 | 14 | 0.982 |
| PVR | 1 | 8 | 0.182 |
| Percutaneous PVR | 0 | 5 | 0.719 |
| RVOT relief | 0 | 3 | 0.681 |
| Radiofrequency pulmonary valvotomy | 0 | 2 | 0.802 |
| supravalvar PS relief | 0 | 1 | 0.962 |
| None | 4 | 8 | 0.001 |
| **NYHA Classification** | | | |
| 1 | 21 | 348 | 0.616 |
| 2 | 3 | 82 | 0.748 |
| 3 | 0 | 0 | - |
| 4 | 0 | 0 | - |
| No data | 3 | 33 | 0.735 |
| **Indication for index PVR** | | | |
| PR | 15 | 437 | - |
| PS | 9 | 19 | - |
| IE | 3 | 7 | - |
| **Surgical information** | | | |
| Total bypass time, mins | 146.4 ± 120.9 | 103.5 ± 58.3 | 0.084 |
| Total cross clamp time, mins | 31.7 ± 41.0 | 41.0 ± 40.0 | 0.245 |
| Valve size | 24.3 ± 2.0 | 24.9 ± 1.9 | 0.123 |
| **Type of valve at the index operation** | | | |
| Homograft, n (%) | 12 (44.4) | 167 (36.1) | 0.625 |
| Mosaic, n (%) | 11 (40.7) | 109 (23.5) | 0.203 |
| Perimount, n (%) | 2 (7.4) | 150 (32.4) | 0.067 |
| Hancock, n (%) | 2 (7.4) | 29 (6.3) | 0.832 |
| Others, n (%) | 0 | 8 (1.7) | 0.416 |
| **Concomitant PA surgery or pre PA stenting** | | | |
| Yes | 3 (11.1) | 74 (16.0) | - |
| No | 24 (88.8) | 389 (84.0) | - |
| **Surgeon at the index operation** | | | |
| Surgeon A, n (%) | 6 (22.2) | 196 (42.3) | - |
| Surgeon B, n (%) | 9 (33.3) | 103 (22.2) | - |
| Surgeon C, n (%) | 6 (22.2) | 63 (13.6) | - |
| Others, n (%) | 6 (22.2) | 101 (21.8) | - |
| **Indication for re-intervention** | | | |
| IE | 10 | NA | - |
| Homograft | 4 |  |  |
| Mosaic | 3 |  |  |
| Perimount | 2 |  |  |
| Hancock | 1 |  |  |
| PR | 8 | NA | - |
| Homograft | 4 |  |  |
| Mosaic | 4 |  |  |
| Perimount | 0 |  |  |
| Hancock | 0 |  |  |
| PS | 8 | NA | - |
| Homograft | 4 |  |  |
| Mosaic | 3 |  |  |
| Perimount | 0 |  |  |
| Hancock | 1 |  |  |
| Other | 1 (Mosaic) | NA | - |
| **Time until re-intervention** | | | |
| Time, y | 6.1 ± 3.4 | NA | - |
| **Type of re-intervention** | | | |
| PVR | 16 | NA | - |
| Percutaneous PVR | 9 | NA | - |
| Balloon valvuloplasty | 1 | NA | - |
| Other | 1　(Heart transplantation) | NA | - |
| Data are mean±SD or number. TOF indicates tetralogy of Fallot; PS, pulmonary stenosis; RVOT, right ventricular outflow tract; PR, pulmonary regurgitation; RV-PA, right ventricle -pulmonary artery; PVR, pulmonary valve replacement; NYHA, New York Heart Association; PA, pulmonary artery; IE, infective endocarditis. | | | |
